# Mechanisms of bisphenol A and its analogs as endocrine disruptors via nuclear receptors and related signaling pathways

**DOI:** 10.1007/s00204-025-04025-z

**Published:** 2025-03-21

**Authors:** Mark Stanojević, Marija Sollner Dolenc

**Affiliations:** 1 Bisafe Doo, 1000 Ljubljana, Slovenia; 2 Faculty of Pharmacy, University of Ljubljana, 1000 Ljubljana, Slovenia

**Keywords:** Bisphenol A, Bisphenol A analogs, Endocrine disruption, Nuclear receptors, Signaling pathways

## Abstract

Bisphenol A (BPA) is a widely used chemical that is slowly being phased out due to its toxic properties. The industry is therefore looking for alternatives in the form of BPA analogs. However, studies have shown that BPA analogs can have comparable or even stronger endocrine and toxic effects than BPA. This review describes various mechanisms and interactions of BPA analogs with individual nuclear receptors. They interfere with downstream signaling pathways not only by binding to the nuclear receptors, but also by various alternative mechanisms, such as altering receptor expression, affecting co-receptors, altering signal transduction pathways, and even epigenetic changes. Further studies are needed to fully investigate the potential synergistic and additive effects that may result. In the search for a less harmful alternative to BPA, affinity to the nuclear receptor may not be the decisive factor. We therefore recommend a different study approach to assess their effects on the endocrine system before new BPA analogs are introduced to the market to protect public health and the environment.

## Highlights

- BPA analogs can have comparable or even stronger endocrine and toxic effects than BPA.
- BPA and its analogs interact with individual nuclear receptors via different mechanisms.
- BPA and its analogs have different potency for endocrine disruption by different mechanisms.

## Introduction

**Fig. 1:**
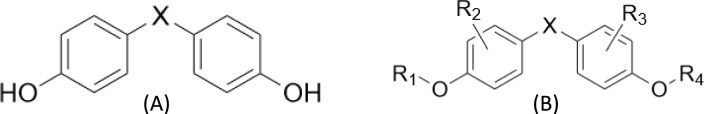
Bisphenol structure presentation. **A** Generic bisphenol structure: two phenyl rings connected by a bridge (X) where X is CH_2_(CH_3_)CH_3_ for BPA, SO_2_ for BPS, CH_2_ for BPF or another group for other bisphenol derivates. **B** Bisphenols can be further derived by attaching the same or different groups to phenol rings (R2, R3) or by substitution of hydroxyl hydrogen with the same or different groups (R1, R4)

Bisphenol A (BPA; 4,4′-isopropylidenediphenol) is a high-volume industrial chemical used extensively in the manufacture of polycarbonate (PC) plastics, epoxy resins and polyvinyl chloride (PVC) plastics, all of which are also used as food contact materials. Global consumption of BPA is estimated at 7.7 million tons in 2022 and is expected to increase at an average annual growth rate of more than 4% over the next few years (GlobalData 2023). As the production of BPA increases, it can be assumed that human exposure to BPA will also increase. However, due to its various adverse health effects on humans (Della Rocca et al. 2023), BPA has been restricted and even banned in certain applications. Canada banned the use of BPA in baby bottles, food packaging and containers in October 2010 (Government of Canada 2010) and the European Union followed suit by restricting the use of BPA in baby bottles in 2011 (Commission Implementing Regulation (EU) No. 321/2011, 2011) and thermal paper in 2016 (Commission Regulation (EU) 2016/2235, 2016). In 2012 and 2013, the US Food and Drug Administration amended its regulations to ban the use of PC resins containing BPA in baby bottles and coatings in infant formula packaging (FDA 2012, 2013). Since October 2013, the US state of Connecticut has banned the use of BPA in thermal receipt paper (State of Connecticut 2011). These restrictions have led to the replacement of BPA in products with other BPA analogs. To date, more than 148 structurally similar substances have been introduced based on the presence of the bisphenol moiety. These include 17 BPA analogs with the generic bisphenol structure and bisphenol derivatives that have components with structural features common to BPA analogs (Fig. 1) (ECHA 2021).

For example, bisphenol S (BPS; 4,40-sulphonyldiphenol), bisphenol B (BPB; 2,2-bis(4-hydroxyphenyl)butane), bisphenol F (BPF; 4,40-dihydroxydiphenylmethane) and bisphenol AF (BPAF; 4,40-(hexafluoroisopropylidene)diphenol) are used in the production of PC plastics and the inner linings of food and beverage cans (Kitamura 2005). BPS is used as a substitute for BPA in thermal papers (Liao et al. 2012). BPF is used in epoxy resins due to its lower viscosity and higher solvent resistance (Geens et al. 2012). BPAF is also used as a crosslinking reagent in fluoropolymers, fluoroelastomers, electronics and optical fibers (Baradie and Shoichet 2005; Konno et al. 2004). Bisphenol Z (BPZ, 4,4′-cyclohexylidene bisphenol) is used to improve the thermal properties and toughness of epoxy resin (Lee et al. 2022). Bisphenol A (BADGE, 2,2-bis(4-(2,3-epoxypropyl)phenyl)propanediglycidyl ether) is the lowest molecular weight oligomer in commercially available epoxy resins and the main component in commercially available liquid epoxy resins (Poole et al. 2004). Tetrabromobisphenol A (TBBPA), tetrachlorobisphenol A (TCBPA) and tetrabromobisphenol S (TBBPS) are brominated flame retardants used in various polymers (Okeke et al 2022; Xu et al 2024; Yang et al. 2020). Other bisphenol A analogs such as bisphenol E (BPE, 4,4′-ethylidenebisphenol), bisphenol AP (BPAP, 4,4′-cyclohexylidenebisphenol), bisphenol P (BPP, 4,4′-phenylidenebisphenol), bisphenol G (BPG, 4,4'-sulfonylbisphenol), bisphenol C (BPC, 4,4′-isopropylidenebis (2-chlorophenol)), bisphenol BP (BPBP, 4,4′-biphenylbisphenol) and bisphenol hexa-epoxide sulfide (BHEPS, hexakis (phenol epoxide) sulfide) are used in polymers to improve material properties (den Braver-Sewradj et al. 2020; Liu et al 2021; Xue et al 2022; Ahn et al 2024; Chen et al 2002; Štampar et al 2023; Russo et al 2018).

For this reason, the consumption of other BPA analogs is also increasing (ChemAnalyst 2024). Demand for BPS was around 185 thousand tons in 2023 and is expected to reach 280 thousand tons in 2033 (ChemAnalyst 2024).

Due to the occurrence of various BPA analogs in the environment, human exposure to these substances is also increasing (Czarny-Krzymińska et al. 2023; Lucarini et al. 2023; Pan et al. 2024). BPA analogs have been shown to have similar or even stronger endocrine and other toxic effects than BPA (Li et al. 2023; Siracusa et al. 2018). In addition, a synergistic and additive toxic effect between BPA and its analogs has also been demonstrated (Zhu et al. 2020). All these effects could be a consequence of different mechanisms and different interactions of different BPA analogs with individual nuclear receptors. The aim of this review is to identify these alternative mechanisms of endocrine disruption by BPA analogs.

## Mechanisms of endocrine system disruption

There is increasing evidence that BPA analogs have an endocrine-disrupting effect through interaction with nuclear receptors. Nuclear receptors are ligand-inducible transcription factors (TFs) that specifically regulate the expression of target genes involved in metabolism, development and reproduction (McKenna et al. 1999). Therefore, BPA analogs can interfere with downstream signaling pathways, which can have multiple adverse effects on organisms. Different BPA analogs may not only interact differently with the same nuclear receptors but also through different pathways that may have an even stronger endocrine-disrupting effect. We will mainly focus on the following:

- Nuclear-receptor binding: endocrine disruptors can bind to hormone receptors and activate or inhibit their activity.
- Membrane receptor binding: endocrine disruptors can bind to membrane hormone receptors but usually activate a different response than nuclear receptors.
- Alteration of receptor expression: endocrine disruptors can affect the expression of hormone receptors in cells by either up- or down-regulating their production.
- Affecting co-receptors: co-regulatory proteins that interact with the hormone receptor can be affected by disruptors, resulting in changes in receptor function.
- Cross-talk with other hormone systems: some disruptors can interfere with the crosstalk between the estrogen receptor (ER) and other hormone systems, such as the aryl hydrocarbon receptor (AhR).
- Modification of signal transduction pathways: endocrine disruptors can alter the signaling pathways downstream of the hormone receptor, affecting the cell’s response to hormones.
- Epigenetic modifications: some disruptors can cause epigenetic modifications, such as DNA methylation, which can affect hormone receptor function and gene expression.

To provide a comprehensive overview of the studies on the endocrine-disrupting effects of BPA analogs, the most important results on their specific interactions with various receptors and signaling pathways are listed in Table 1. The effects of BPA analogs, mediated through nuclear receptors or other signaling pathways, are described in more detail in the individual chapters below.

**Table 1:** Sumary of studies on BPA and its analogs

Study	BPA analog	Effect	Pathway modulated
Steinmetz et al. (1997)	BPA	Weak ERα and ERβ agonist compared to E2	Estrogen receptor signaling
Kojima et al. (2019)	BPAF, BPB, BPZ, BPA, BPE, BPF, BPS, BPAP, BPP	ERα/β agonistic and antagonistic activity ranking among analogs	Estrogen receptor signaling
Nadal et al. (2018)	BPA and analogs	Challenges concept of BPA as weak estrogen	Estrogen receptor signaling
Alonso-Magdalena et al. (2006)	BPA	Rapid non-genomic effects on pancreatic β-cells	Membrane estrogen receptor signaling
Ziv-Gal et al. (2013)	BPA	Minor effect	AhR signaling
Shan et al. (2023)	BPA, BPS, TBBPA	Activating AhR signaling	AhR signaling
Nadal et al. (2000)	BPA	Induced Ca^2+^ signaling	Ca^2+^ signaling
Rehfeld et al. (2020)	BPA BPG, BPAF, BPC, BADGE, BPB, BPBP	No effect Activates CatSper Ca^2+^ channel in sperm	Ca^2+^ signaling
Doshi et al. (2011)	BPA	Epigenetic modifications of ERα and ERβ in testes	Epigenetic modifications, DNA methylation
Bhandari et al. (2019)	BPA	Esr1 and Esr2 methylation, aromatase increase	Epigenetic modifications, DNA methylation
Du & Taylor (2004)	BPA	Hoxa10 methylation, embryonic uterine development	Epigenetic modifications, DNA methylation
Khodasevich et al. (2024)	BPA	GRIK1 methylation, linked to obesity	Epigenetic modifications, DNA methylation
Castillo Sanchez et al. (2016)	BPA	Activation of MMPs and the GPER/ EGFR/ERK1/2 signaling pathways	GPER signaling
Sheng et al. (2013)	BPA	GPER-mediated cell proliferation in germ cells	GPER signaling
Lei et al. (2021a; b)	BPAF	Activates PI3K/Akt via GPER1	GPER signaling
Lei et al. (2021a, b)	TCBPA	Activates PI3K/Akt via GPER1	GPER signaling
Yu et al. (2023)	TCBPA, BPAF	Induced migration of human breast cancer cells SK-BR-3	GPER signaling
Dong et al. (2021)	BPA	TGF-β signaling, cell proliferation effects	TGF-β signaling pathway
Jia et al. (2018)	BPS	ERRα promotes invasion via fibronectin regulation	ERRα signaling
Liu et al. (2019)	BPA	Binding activity similar to natural ligands	ERRγ signaling
Okada et al. (2008)	BPAF, BPF, BPAP, BPB, BPA, BPE	ERRγ binding activity ranking among analogs	ERRγ signaling
Zou et al. (2022)	BPA	Sex-specific ERRγ signaling alternation	ERRγ signaling
Song et al. (2015)	BPA	ERRγ upregulation, proliferation of cancer cells	ERRγ signaling
Dong et al. (2021)	BPA	Proposed TGF‑β signaling pathway	ERRγ signaling
Jia et al. (2018)	BPS	ERRα and FN1 promoter binding promotion, tumor progression	ERRα signaling
Grimaldi et al. (2019)	BPCcl	most potent antiandrogenic activity	AR signaling
Paris et al. (2002)	BPA	Weak antiandrogen	AR signaling
Kojima et al. (2019)	BPA, BPE, BPB, BPF, BPZ, BPP, BPAP, BPAF	AR antagonistic activity varies by analog	AR signaling
Wang et al. (2017)	BPA	Preventing AR dimerization, promoting AR and co-repressors interactions	AR signaling
Sheng et al. (2012)	BPA	Recruits NCoR/SMRT, suppresses TR transcription	TR signaling
Hu et al. (2023)	BPA, BPS, TBBPA, TBBPS	Alters thyroid hormone synthesis genes	TR signaling
Prasanth et al. (2010)	BPA	Binding energy similar to known GR antagonist	GR signaling
Sargis et al. (2010)	BPA	increase in GR-mediated luciferase expression	GR signaling
Kolšek et al. (2015)	BPA, BPF, BPZ, BHEPS	GR antagonistic and agonistic activities identified	GR signaling
Kojima et al. (2019)	BPAF, BPP, BPAP, BPB, BPZ, BPA	GR antagonistic activity ranking among analogs	GR signaling
Kitraki et al. (2015)	BPA	Epigenetic modification of GR co-regulator FKBP5	GR signaling
Ahmed and Atlas (2016)	BPA, BPS	PPARγ activation	PPARγ signaling
Fang et al. (2015)	TBBPA, TCBPA	PPARγ activation	PPARγ signaling
Riu et al. (2011)	BPA, BPF	PPARγ activation	PPARγ signaling
Zhang et al. (2018)	BPA, BPC, BPAF, TBBPA, TCBPA	PPARα agonistic activity by halogenated BPAs	PPARα signaling
Li et al. (2021)	BPA, BPS, BPAF, BPF, BPB, TBBAP, TCBP	PPARβ/δ agonistic activity	PPARβ/δ signaling
Molina-Molina et al. (2013)	BPA, TCBPA, TBBPA, BPS, BPF	PXR activation ranking among analogs	PXR signaling
Sui et al. (2012)	BPA	species-specific effects	PXR signaling
Sui et al. (2014)	BPA	species-specific effects	PXR signaling

### Influence of BPA and its analogs on estrogen activity

The best-studied mechanism of the endocrine effect of BPA is estrogen signaling. Estrogen receptors (ER) play a crucial role in orchestrating the effects of estrogens on various physiologic processes. These processes include the development and operation of the female reproductive system. As a steroid, endogenous estrogens can freely enter a cell and interact with various receptors. The most important and well-studied mechanism is the interaction with ERs, which ultimately leads to changes in gene transcription (Chen et al. 2022; Marino et al. 2006). The ability of BPA to bind ERα and ERβ is extremely weak, with a 10,000-fold lower affinity than 17b-estradiol (E2) for both ER subtypes. The estrogenic potency of BPA determined in vitro was between 1000- and 5000-fold lower than that of E2 (Steinmetz et al. 1997) and doses in the micromolar range (> 1 µM) are required to achieve an estrogenic effect. Compared to BPA, the rank order of ERα agonistic activity induced by nine BPA analogs studied was BPAF > BPB > BPZ > BPA, BPE, BPF > BPS > BPAP > BPP. The order of ER β-agonist activity induced by these compounds was similar: BPAF > BPZ > BPB > BPE, BPA > BPF > BPAP > BPS. However, even the affinity of the potent ERα- and ERβ- agonist (BPAF) was only about tenfold stronger than that of BPA. On the other hand, BPAF and BPP have been found to act as ERα and ERβ antagonists at lower concentrations (Kojima et al. 2019). However, in recent years, an increasing number of studies have shown that BPA analogs can induce estrogen-like effects with the same potency as E2, challenging the concept of BPA and analogs as weak estrogens that have no effect at low doses (Nadal et al. 2018). To understand this discrepancy, we should note that estrogens do not only act by binding to ERα and ERβ, migrating to the nucleus and acting as TFs that bind to EREs.

**Fig. 2:**
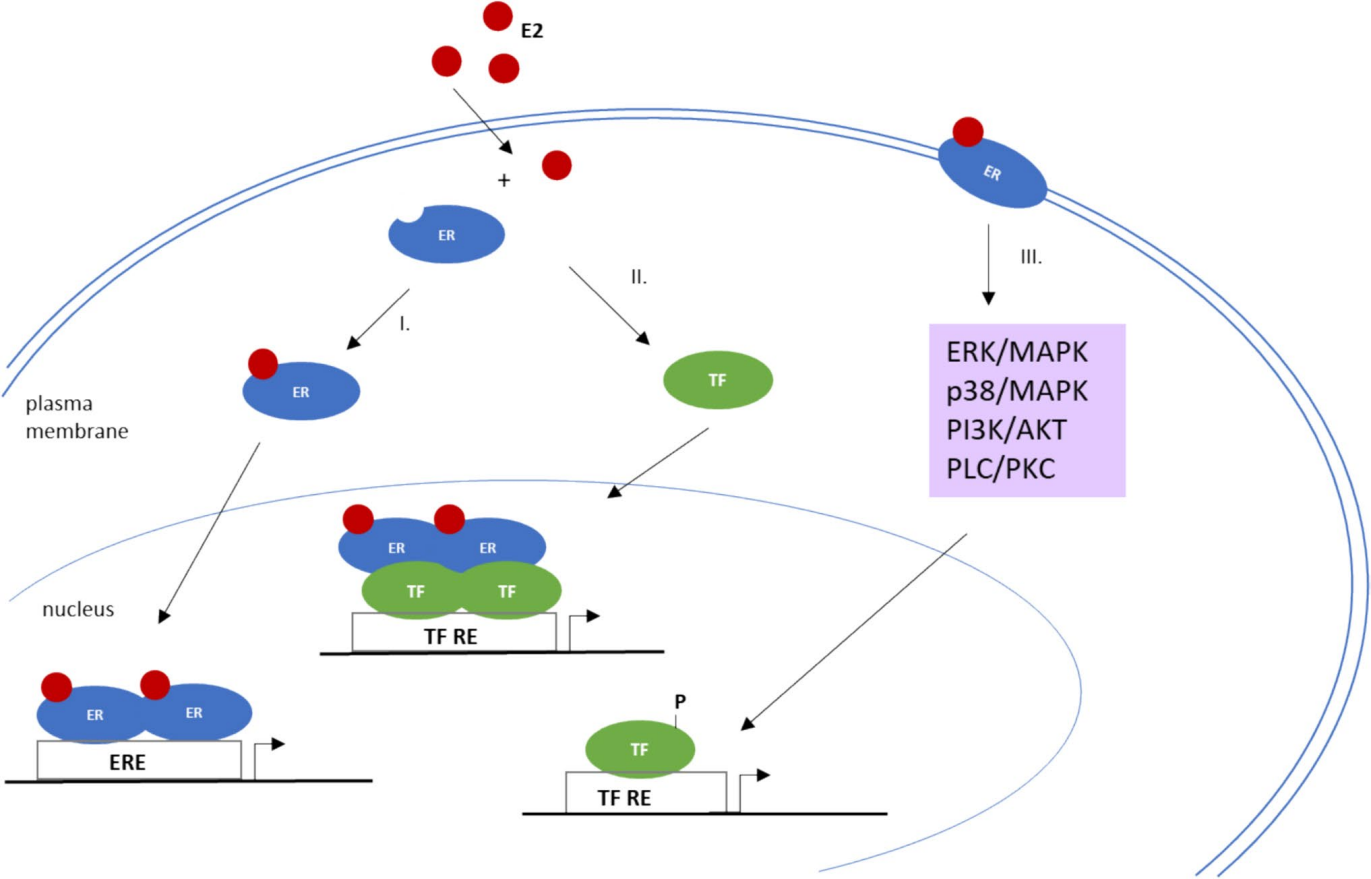
The representation of different mechanisms of estrogen signaling (adapted from Vrtačnik et al. 2014). I. The direct genomic pathway, which is considered the classical mechanism of estrogen signaling, promotes expression of the target gene by direct binding of the E2-ER dimer to the ERE. II. In the indirect genomic signaling pathway, E2-activated ERs bind DNA through protein–protein interactions with other classes of transcription factors at their respective response elements. III. The non-genomic signaling pathway begins with the binding of E2 to ERs located at the plasma membrane, which leads to the activation of various protein kinase cascades. These can ultimately lead to changes in gene expression via the phosphorylation of transcription factors. *E2* 17β-estradiol; *ER* estrogen receptor; *ERE* estrogen response element; *P* phosphate group; TF, transcription factor; TF RE, transcription factor response element; *ERK* extracellular signal-regulated kinases; *MAPK* mitogen-activated protein kinase; *Pi3K* phosphoinositide 3-kinase; *AKT* protein kinase B; *PLC* phospholipase C; *PKC* protein kinase C

Endogenous estrogens also modulate gene expression via a second indirect mechanism in which the ER interacts with other TFs such as activator protein (AP)−1, nuclear factor-(B) (NF-(B) and stimulatory protein-1 (Sp-1) by stabilizing DNA–protein complexes and/or recruiting co-activators. In addition, binding of estrogen to the ER can also trigger a rapid action that begins with the binding of E2 to ERs located at the plasma membrane and leads to the activation of various protein kinase cascades (e.g., ERK/MAPK, p38/MAPK, PI3K/AKT, PLC/PKC). These can ultimately lead to changes in gene expression through the phosphorylation of TFs (Fig. 2) (Marino et al. 2006).

Mechanisms involving the aryl hydrocarbon receptor (AhR) can also promote or inhibit ER signaling. The two signal transduction pathways interact via different mechanisms in the originally described inhibitory AhR-ER crosstalk. Although they do not rule out alternative processes, recent studies suggest rapid proteasome-dependent degradation of the ER (Safe and Wormke 2003). In contrast, (Ohtake et al. 2003) proposed a model of crosstalk between AhR and unliganded ER in which the ligand-bound AhR acts as a co-activator of ER signaling. These results were supported by extensive in vitro and in vivo studies. The extensive list of AhR agonists that exhibit estrogenic activity includes chlorinated aromatic compounds (2,3,7,8-tetrachlorodibenzo-p-dioxin (TCDD), 3,39,4,49,5-pentachlorobiphenyl (PCBP), 3,39,4,49-tetrachlorobiphenyl (TCBP)), PAHs (3-methyl-cholanthrene (3MC) and benzo(a)pyrene (BaP)), heteroaromatics (indolo[3,2-b]carbazole), indole dimers (DIM), chrysin and other flavonoids that act as AhR agonists and antagonists (Liu et al 2006). However, the AhR-signaling pathway has been shown to play a minor role in mediating the adverse effects of BPA (Ziv-Gal et al. 2013). However, Shan et al. demonstrated that exposure to environmentally relevant concentrations of bisphenols (BPA, BPS, TBBPA) at 1 µM can disrupt the expression of crucial molecules involved in oxidative stress and neuronal function by activating the AhR-signaling pathway, leading to neurotoxicity (Shan et al. 2023).

In addition to genomic mechanisms, the membrane ER is also involved in non-genomic disruption of homeostasis through Ca^2+^ signaling. Although the pancreas is not usually considered a classical estrogen target, estrogen influences insulin and glucagon secretion in islet cells through both the nuclear and membrane ER (Nadal 2004). Ca^2+^ ion oscillations have been described as finely regulated by E2 in both α- and β-cells. In glucagon-containing α-cells, E2 causes suppression of Ca^2+^ ion oscillations generated by low glucose, whereas the gonadal hormone potentiates Ca^2+^ ion signaling in β-cells. In both cell types, E2 action is triggered after binding to a membrane-bound ER (Nadal et al. 2000; Ropero et al. 2002). They demonstrated that low doses of BPA (1 nM) in the pancreatic islets of Langerhans of mice induced rapid, non-genomic changes in Ca^2+^ behavior. Physiologic concentrations of E2 enhanced Ca^2+^ signaling and insulin release in freshly isolated mouse islets of Langerhans. BPA mimicked E2 at exactly the same concentrations (100 pM–1 nM) (Nadal et al. 2000). The rapid effect of BPA in the endocrine pancreas was not limited to the β-cells. BPA rapidly inhibited low glucose-induced intracellular Ca^2+^ oscillations in mouse pancreatic α-cells. Moreover, the effects of low doses of BPA were observed not only in freshly isolated cells from islets of Langerhans, but also in vivo. A single injection of 10 µg/kg body weight BPA resulted in a rapid decrease in blood glucose levels and an increase in plasma insulin (Alonso-Magdalena et al. 2006). No data are available on the influence of other bisphenol analogs on Ca^2+^ signaling in islet cells. However, BPA analogs can activate cation channel of sperm (CatSper) and thereby affect Ca^2+^ signaling in human sperm cells. The CatSper is the most important Ca^2+^ channel in human spermatozoa (Lishko et al. 2011) and is activated by the female sex steroid progesterone. The rank order of potency was BPG > BPAF > BPC > BADGE > BPB > BPBP. Interestingly, BPA did not activate the CatSper in human sperm (Rehfeld et al. 2020).

BPA is also associated with epigenetic changes in the promoter region of ERα and Erβ. Doshi et al. conducted a study showing that exposure of male rats to a daily dose of 2.4-µg BPA during the neonatal period resulted in excessive methylation of the promoter region of ERα and ERβ in their testes throughout maturation. This suggests that BPA has an epigenetic effect by causing abnormal DNA methylation. In addition, they also found an increase in the expression of Dnmt3a and Dnmt3b at both transcript and protein levels. These de novo DNA methyltransferases are enzymes responsible for DNA methylation. Given that BPA has estrogenic properties, it is hypothesized that the upregulation of Dnmts expression in adulthood due to BPA exposure occurs via an ER-dependent pathway (Doshi et al. 2011).

Similarly, Bhandari et al. demonstrated that in utero exposure of developing mouse embryos to a BPA dose of 50 µg/kg/day alters the expression of the genes Esr1, Esr2, aromatase and DNA methyltransferase in mesenchymal cells in the developing proximal urogenital sinus of fetal male mice. Methylation of selected CpGs in the CpG island of Esr1 exon 1A and Esr1 exon 1C by in utero exposure to BPA. Methylation of the CpG island of the Esr2 promoter was also significantly increased. Global DNA methylation levels also showed a similar pattern to Esr1 promoter methylation, suggesting that BPA exposure causes hypermethylation not only of ER genes but also of many other genes in the genome of developing fetal mesenchymal cells. Interestingly, E2 at doses equivalent to those in mixed oral contraceptives (0.4 µg/kg/day) caused many, but not all, of these effects, suggesting that some effects of BPA may not be solely due to its known estrogenic activity. Furthermore, since BPA can serve as an agonist for estrogen receptors, a BPA-induced increase in aromatase would lead to increased estradiol levels in estrogen-target mesenchymal cells expressing Esr1 during fetal life, resulting in a “double hit” of additional estrogen, both exogenous (BPA) and endogenous (estradiol) (Bhandari et al. 2019).

It has also been shown that BPA exposure in utero leads to aberrant methylation in the promoter and intron of Hoxa10, which persists after birth. Hoxa10 plays a critical role in embryonic uterine development (Du and Taylor 2004) and is also expressed in the adult endometrium, where it is involved in implantation and regulated by sex hormones (Daftary and Taylor 2000; Eun Kwon and Taylor 2004). However, there were no significant changes in DNA methylation of Hoxa10 in individuals treated intraperitoneally with BPA. This finding suggests that epigenetic changes, i.e., changes in DNA methylation, may only occur within a specific and crucial developmental period (Doherty et al. 2010).

This suggests that methylation-mediated epigenetic changes may be one of the possible pathways through which BPA negatively affects spermatogenesis and fertility.

A study in humans confirms associations between prenatal exposure to environmental BPA (about 1.3 µg/L) and DNA methylation at birth, as well as differentially methylated CpG sites and differentially methylated regions throughout the epigenome. Sex-specific associations between DNA methylation and response to prenatal BPA exposure were demonstrated. In addition, several CpGs remained associated with prenatal BPA exposure into adolescence, demonstrating the persistence of changes in DNA methylation. The most prominent methylation probes in the male BPA models showed a statistically significant positive correlation with a CpG site in the *GRIK1* gene (Khodasevich et al. 2024). This site encodes a subunit of the ionotropic glutamate receptor kainate 1 (GRIK1), which belongs to the kainate family and is involved in excitatory neurotransmission in the central nervous system (Negrete-Díaz et al. 2022). A previous epigenome-wide association study conducted on peripheral blood from a group of older adults with an average age of over 60 years showed a remarkable association between DNA methylation in GRIK1 and body mass index (BMI) (Sayols-Baixeras et al. 2017). In addition, an association between BPA exposure and a higher likelihood of obesity was demonstrated (Naomi et al. 2022), underlining the reciprocal positive relationships between BPA and BMI in the presence of methylation in GRIK1.

As mentioned above, the classical genomic effect of BPA and its analogs should not be considered as the main mechanism of endocrine disruption. A re-examination of human bioburden from bisphenol A in European women showed that the geometric mean of total urinary BPA concentrations in the participating studies ranged from 0.77 to 2.47 µg/L (3.4–10.8 nM) (Tagne-Fotso et al. 2024). A similar BPA concentration of around 1.3 µg/L (5.7 nM) was measured in the umbilical cord blood of newborns (Khodasevich et al. 2024). At such low levels of BPA exposure, it is more likely that the alternative mechanisms cause endocrine disruption related to estrogen signaling.

### Signaling activity of BPA and its analogs through G-protein-coupled estrogen receptor

**Fig. 3:**
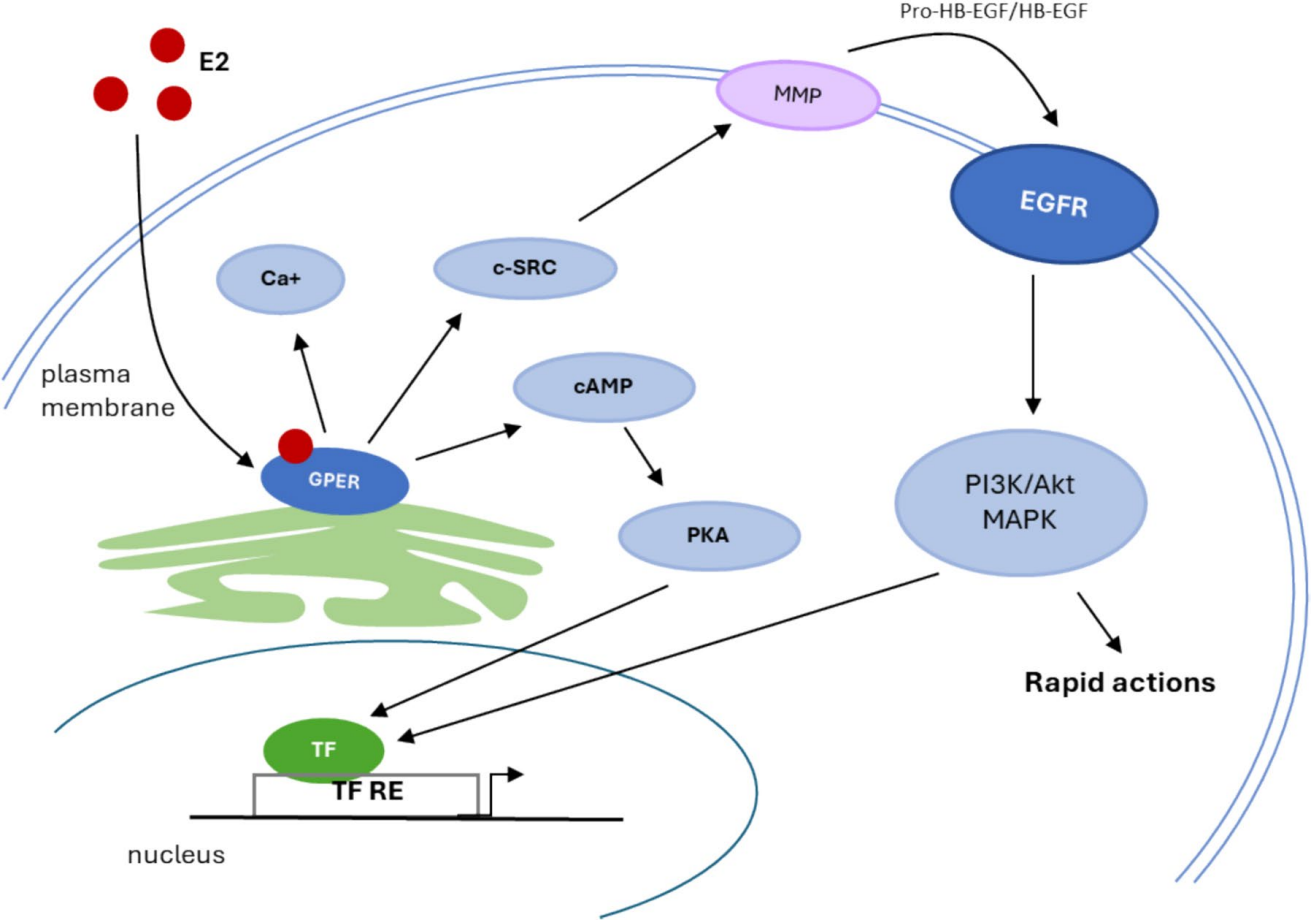
GPER signaling pathways. Activation of GPER stimulates the production of cAMP, the mobilization of calcium and c-Src. Elevated intracellular cAMP levels activate PKA, which leads to transcriptional regulation via phosphorylation of CREB. C-Src activates the MMPs. These MMPs cleave proHBEGF and release free HBEGF, which transactivates EGFR, which in turn activates MAPK and PI3K/Akt signaling pathways that can trigger additional rapid (non-genomic) effects or genomic effects that regulate gene transcription. *E2* 17β-estradiol; *EGFR* epidermal growth factor receptor; *GPER* G protein-coupled ER; *MMP* matrix metalloproteinase; *HB-EGF* heparin-binding epidermal growth factor; *TF* transcription factor; *PKA* protein kinase A; *CREB* cAMP response element-binding protein; *MAPK* mitogen-activated protein kinase; *PI3K* phosphoinositide 3-kinase; *Akt* protein kinase B

In 2005, the orphan receptor GPR30 was characterized as a G-protein-coupled estrogen receptor (GPER) that is localized pronominally at the endoplasmic reticulum, and only minor amounts are present on the plasma membrane (Cheng et al. 2011). The signal transmission by GPER takes place via the transactivation of the epidermal growth factor receptor (EGFR) and with the involvement of non-receptor tyrosine kinases of the Src family (c-Src). In this mechanism, which is now also accepted for other G-protein-coupled receptors, stimulation of GPER activates metalloproteinases and induces the release of heparin-binding epidermal growth factor (EGF), which binds and activates EGFR, leading to activation of downstream signaling molecules, leading to the activation of mitogen-activated protein kinase MAPK and phosphoinositide 3-kinase (PI3K)/protein kinase B (Akt) pathways that can induce additional rapid (non-genomic) effects or genomic effects regulating gene transcription. In addition to the rapid signaling events mentioned above, GPER also indirectly regulates transcriptional activity through the activation of signaling mechanisms involving cyclic adenosine monophosphate (cAMP) and intracellular calcium mobilization (Fig. 3) (Prossnitz and Barton 2011). Increased intracellular cAMP levels, activate protein kinase A (PKA), which leeads to transcriptional regulation via the phosphorylation of cAMP response element-binding protein (CREB) (Girgert et al. 2019)**.** Accumulating data suggests that the GPER-mediated signaling pathway is closely related to cancer cell growth, survival, proliferation, migration and invasion following BPA treatment (Murata and Kang 2018). BPA upregulates matrix metalloproteinase-2 and −9 (MMPs) through GPER, not ER, and also induces activation of extracellular signal-regulated kinases (ERK) 1 and 2 through GPER/epidermal growth factor receptor (EGFR) in lung cancer cells. These results suggest that BPA triggers lung cancer cell migration and invasion through the activation of MMPs and the GPER/EGFR/ERK1/2 signaling pathways (Zhang et al. 2014). In addition, BPA-induced migration of ER-negative breast cancer cells through the GPER signaling pathway depends on the activation of focal adhesion kinase (FAK), Src and ERK2 and a subsequent increase in Activator Protein-1 (AP-1)/nuclear factor κ-light-chain-enhancer of activated B cells (NF-κB) DNA-binding activity (Castillo Sanchez et al. 2016). The GPER/EGFR/ERK signaling pathways may also be involved in BPA-mediated initiation and progression of male germ cell carcinogenesis.

BPA at an environmentally relevant low concentration (1 nM) induces GPER expression by activating the GPER-mediated EGFR-ERK-ER-α-c-Fos pathway, forming a stimulatory feedback loop that further enhances BPA-induced proliferation of mouse spermatogonial germ cells (GC-1). These data highlight the effect of BPA in the initiation and progression of cancer, thereby providing strong support to the growing recognition of the adverse effects of BPA on human reproductive health (Sheng et al. 2013).

In addition, there is increasing evidence that other BPA analogs mimic the mode of action of BPA. Studies suggest that BPS acts both as a weak estrogen by binding directly to the ER (Li et al. 2018) and as a strong estrogen when acting via extranuclear localized ERs or GPER (Viñas and Watson 2013). BPAF and BPB showed a much stronger agonistic and promoting activity to GPER than BPA (Cao et al. 2017). In addition, Yu et al. demonstrated that tetrachlorobisphenol A (TCBPA) and BPAF induce the activation of PI3K/Akt and its downstream signaling targets by GPER1-EGFR at the mRNA level (Yu et al. 2023).

The expression levels of 18 target genes, including GPER1, EGFR, MAPK, Akt and related downstream genes, were determined by qRT-PCR to determine how low levels of BPAF exposure affect GPER1-regulated Erk and PI3K/Akt signaling pathways. The target genes play a role in the proliferation of breast cancer cells and many of them are also genetic factors that increase the likelihood of developing breast cancer. Exposure to 0.001-μM BPAF resulted in upregulation of 17 target genes based on mRNA levels compared to control cells, while all target genes were significantly increased upon exposure to 0.01 and 0.1-μM BPAF (Lei et al. 2021a). 17 target genes were analyzed for expression after exposure to TCBPA. 16 genes showed increased expression at the mRNA level when exposed to 1-μM TCBPA compared to control cells, and all genes showed significant upregulation after 10-μM TCBPA exposure (Lei et al. 2021a). This activation also affects the migration of human breast cancer cells SK-BR-3 induced by TCBPA and BPAF (Yu et al. 2023). Their previous studies also showed that BPAF at a concentration of 0.01 μM and TCBPA at a concentration of 1 μM increase the expression of GPER1 protein, leading to increased phosphorylation of Akt and ERK1/2. These results provide further confirmation of the involvement of PI3K/Akt signaling pathways via GPER1 in the regulation of SK-BR-3 cell migration induced by BPAF and TCBPA (Lei et al. 2021b, b).

The toxic effect of BPA and analogs via GPER is a good example of how the response can be amplified by the activation of different signaling pathways and stimulatory loops. Therefore, the concentrations of BPA and analogs required for a toxic effect via GPER are lower than for the classical genomic pathways via nuclear receptors. In addition, some BPA analogs, as with ER, have a stronger toxic effect than BPA via GPER. Consideration should therefore be given to including the GPER pathway in the testing of new BPA analogs before they are marketed.

### Endocrine disruption of BPA and its analogs mediated by estrogen-related receptors

Estrogen-related receptors (ESRRs) form a subgroup within the nuclear receptor family NR3B, which comprises three different members: estrogen-related receptors alpha (ESRRα), beta (ESRRβ) and gamma (ESRRγ) (Tremblay and Giguère 2007). ESRRα and ESRR (are predominantly expressed in metabolically active tissues that preferentially use fatty acids as a primary energy source, such as the heart, brown adipose tissue, cerebellum, intestine and liver (Bookout et al. 2006; Sladek and Giguère 1999; Xia et al. 2019). The physiologic functions of ERRs have been extensively studied in recent years and it is now widely agreed that these receptors play a crucial role in monitoring cellular energy metabolism. This is evidenced by their increased expression in tissues with high energy demands and their ability to influence a broad spectrum of metabolic genes (Deblois and Giguère 2013). In particular, ERRs play a role in the regulation of genes associated with various aspects of mitochondrial function, including the Krebs cycle, as well as lipid, carbohydrate, pyruvate, amino acid and nucleic acid metabolism (Eichner and Giguère 2011). In addition to their central role in cellular energy metabolism, research has shown that ERRs contribute to the regulation of circadian rhythm, cardiac, renal and skeletal muscle physiology, and cell growth and differentiation (Ranhotra 2012). It appears that ERRα and ERRγ together control the oxidative metabolic system in the heart (Dufour et al. 2007).

The interplay between ESRRs and estrogen signaling involves reciprocal transcriptional regulation or reciprocal binding to each other’s response elements in common target genes, which occurs in a context-specific manner (Audet-Walsh and Giguére 2015). Although ERRs are considered constitutively active orphan receptors, cholesterol has recently been shown to act as an endogenous ERRα agonist (Casaburi et al. 2018; Wei et al. 2016). On the other hand, some exogenous molecules such as BPA show strong specificity as an agonist for the ERRγ (Brieno-Enriquez et al. 2012; Héliès-Toussaint et al. 2014; Song et al. 2015; Tohmé et al. 2014), with a binding affinity 800- to 1000-fold higher than on other receptors (Takayanagi et al. 2006). Recent research suggests that the binding of BPA to ERRγ is very similar to the interaction with natural ERRγ ligands (Liu et al. 2019). Short-term exposure to BPA induces the expression of ERRγ mRNA and protein in various cell lines, including those of lung cancer, breast cancer, adipocytes, hepatocytes and zebrafish (Héliès-Toussaint et al 2014; Ryszawy et al 2020; Song et al 2015; Tohmé et al 2014; Zhang et al. 2016). In addition, a recent study suggests that exposure to a physiologically relevant concentration (1 μM) of BPA alters ERRγ signaling pathways in human placental explants in a sex-specific manner. This suggests that BPA may induce or enhance placental dysfunction via ERRγ and contribute to the pathophysiology of fetal growth restriction (Zou et al. 2022). While there is not much evidence on how BPA analogs affect ERRγ signaling, they have been shown to have an even stronger binding affinity for ERRγ. The rank order was BPAF > BPF > BPAP > BPB > BPA > BPE (Okada et al. 2008).

However, we are still in the dark as to the nature and extent to which BPA analogs may affect the physiologic functions of ERRγ. It is hypothesized that upregulation of ERRγ, but not GPER and ERα/β, plays an important role in BPA-mediated proliferation of breast cancer cells. ERK1/2 is involved in BPA-induced upregulation of ERRγ, as shown by the significantly reduced upregulation of ERRγ and proliferation of breast cancer cells in the presence of an ERK1/2 inhibitor (Song et al. 2015).

**Fig. 4:**
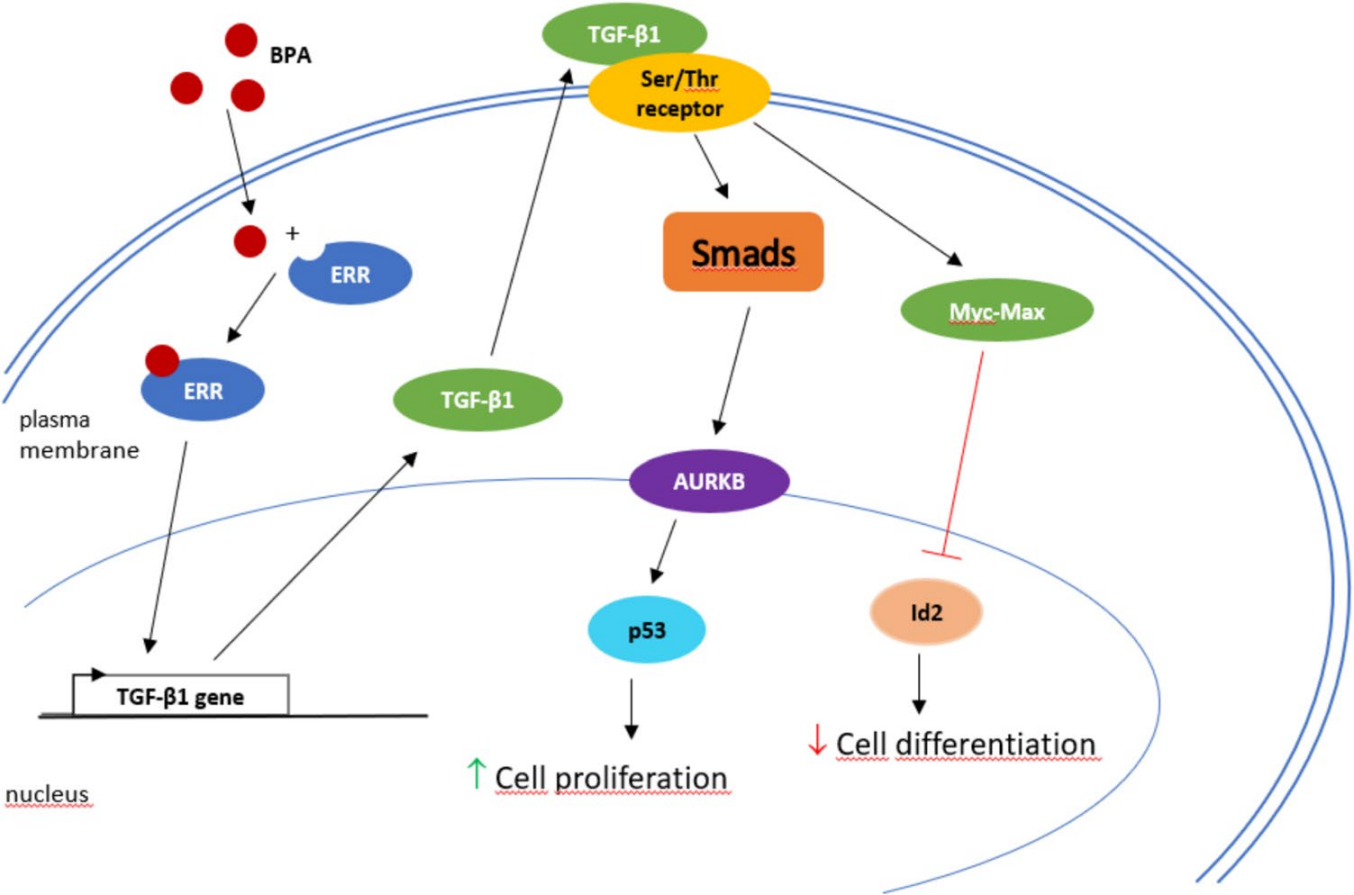
The BPA disruption mechanism via the TGF‑β signaling pathway (adapted from Dong et al. 2021). Upon BPA exposure, the TGF‑β1 signaling pathway is activated via the ERRα‑BPA complex bound to the TGF‑β1 gene promoter, leading to cell proliferation and inhibition of cell differentiation. *BPA* bisphenol A; *ERR* estrogen-related receptor; *TGF‑β1* transforming growth factor-β1; *AURKB* aurora kinase B; *Myc-Max* Myc/Max heterodimer; *Id2* inhibitor of DNA binding 2; *Smads* suppressor mothers against decapentaplegia

In contrast, Dong et al. (2021) proposed a detailed mechanism of BPA regulation by the transforming growth factor beta 1 (TGF‑β) signaling pathway (Fig. 4), which is triggered by the ERRα-BPA complex. The signaling pathway is triggered by the binding of TGF‑β1 to the serine/threonine kinase receptors on the cell surface and then relayed by the intracellular mediators, known as Smads (Suppressor mothers against decapentaplegic). Activation of Smads leads to their translocation from the cytoplasm to the nucleus, where, together with TFs, they activate or repress transcription to regulate the expression of target genes (Attisano and Tuen Lee-Hoeflich 2001). Aurora kinase B (AURKB) and inhibitor of DNA binding 2 (Id2) are downstream TFs of this signaling pathway that promote cell proliferation and inhibit cell differentiation (He et al. 2019; Siegel et al. 2003). The results of the study confirmed that BPA can significantly decrease the expression of Id2 and increase the expression of AURKB, resulting in human neural stem cell proliferation and inhibition of cell differentiation (Dong et al. 2021), confirming the proposed mechanism.

Furthermore, BPS at nanomolar concentrations has been shown to be able to induce the migration and invasion of pheochromocytoma PC12 cells through ERRα-mediated regulation of fibronectin and the miR-10b/KLF4 axis. Mechanistically, BPS can increase the expression of fibronectin (FN1), a robust mesenchymal marker of cancer cells, by promoting the binding between ERRα and the FN1 promoter. At the same time, BPS can upregulate miR-10b, a known pro-metastatic microRNA, in an ERRα-dependent manner. The increased levels of miR-10b lead to suppression of Krüppel-like factor 4 (KLF4), a tumor suppressor and transcription factor involved in the maintenance of epithelial cell differentiation and inhibition of epithelial-to-mesenchymal transition (EMT). Suppression of KLF4 interferes with cell cycle control and differentiation programs, which further promotes PC12 cell migration and invasion. In addition, BPS contributes to the upregulation of both mRNA and protein levels of ERRα, reinforcing its role in the transcriptional activation of FN1 and miR-10b. This process is coupled with the enhanced nuclear translocation of ERRα, allowing it to exert greater transcriptional control over genes involved in cell migration and invasion. In addition, the integrin-linked kinase (ILK) signaling pathway, which is known to interact with fibronectin, may further enhance the pro-migratory and invasive properties of PC12 cells. Focal adhesion kinase (FAK), another key effector downstream of fibronectin-integrin interactions, could also be activated, leading to phosphorylation of downstream targets such as AKT and ERK, which are important regulators of cell survival, motility and cytoskeletal reorganization. Altogether, these results highlight the role of BPS in promoting an invasive phenotype in PC12 cells via the ERRα-FN1 and miR-10b/KLF4 signaling networks. This suggests a possible mechanism by which environmental endocrine disruptors may contribute to tumor progression and metastasis (Jia et al. 2018).

The studies presented here show that the interaction of ERRs with BPA and its analogs may play an important role in disrupting the endocrine system, leading to adverse health effects. As there are still gaps in the understanding of the mechanisms of action and adverse effects, further studies should be conducted. It is important to define the mechanisms and toxicity not only for BPA but also for its analogs. As shown, different BPA analogs have toxic effects via EERs at different concentrations via different mechanisms.

### Influence of BPA and its analogs on androgen activity

Androgens and the androgen receptor (AR) play a crucial role in male reproduction. The biologic functions of androgens are primarily mediated by AR signaling. Upon binding to endogenous androgens, the AR undergoes a conformational change, dimerizes, and trans-locates from the cytoplasm to the nucleus to bind androgen response elements (ARE) and thus regulate gene transcription (Hartig et al. 2011). AR activation can be suppressed by some ECDs, including BPA analogs. Among BPA analogs, chlorinated bisphenol C (BPCcl) showed the most potent antiandrogenic activity (IC50 = 171 nM) using an AR reporter cell line, which has the same potency as the vinclozolin metabolite M2, which is the most potent xenoantiandrogen known (Grimaldi et al. 2019). On the other hand, BPA has a relatively low affinity (IC50 = 7 µM) for AR, so it is considered a weak antiandrogen (Paris et al. 2002). The study, which investigated the influence of BPA and its analogs on nuclear receptors using in vitro transactivation assays, found that most other BPA analogs have an even lower affinity for AR. The ranking of the other BPA analogs compared to BPA was BPE > BPB, BPA > BPF > BPZ, BPP, BPAP > BPAF. However, the affinity of BPE was still estimated to be 15 times lower than the affinity of hydroxyflutamide, a known AR antagonist (Kojima et al. 2019). A recent study, in which the interaction of BPA and its analogs with AR was investigated using computational methods, showed that the four BPA analogs (Pergafast 201, 2,2-Bis(2-hydroxy-5-biphenylyl)propane (BPPH), Benzenesulfonamide, *N,N'*-(methylenebis(4,1-phenyleneiminocarbonyl))bis(4-methyl (BTUM), and 4-((4-(Benzyloxy)phenyl)sulfonyl)phenol (BPSP)) investigated have a higher binding affinity (− 10.2 to − 8.7 kcal/mol) to AR than BPA (− 8.6 kcal/mol). However, further experimental validation of the results showed that BPA has higher anti-androgenic activity compared to the other selected compounds. The study showed that only BPA disrupted dihydrotestosterone (DHT) induced AR dimerization. This is due to the binding behavior of BPA to AR, which is similar to the binding behavior of DHT, while the other BPA analogs have different binding properties (Pathak et al. 2024).

In the presence of androgens, the amino-terminal (N-terminal) and carboxyl-terminal (C-terminal) domains of AR interact, a process known as N/C-terminal interaction. This intramolecular interaction is crucial for stabilizing AR and promoting its dimerization, which is essential for its function as a transcription factor (Centenera et al. 2008). However, BPA antagonizes AR signaling by disrupting this N/C interaction, which in turn prevents AR dimerization and reduces the stability of AR (Wang et al. 2017). This destabilization impairs the ability of AR to bind to DNA and regulate gene expression.

**Fig. 5:**
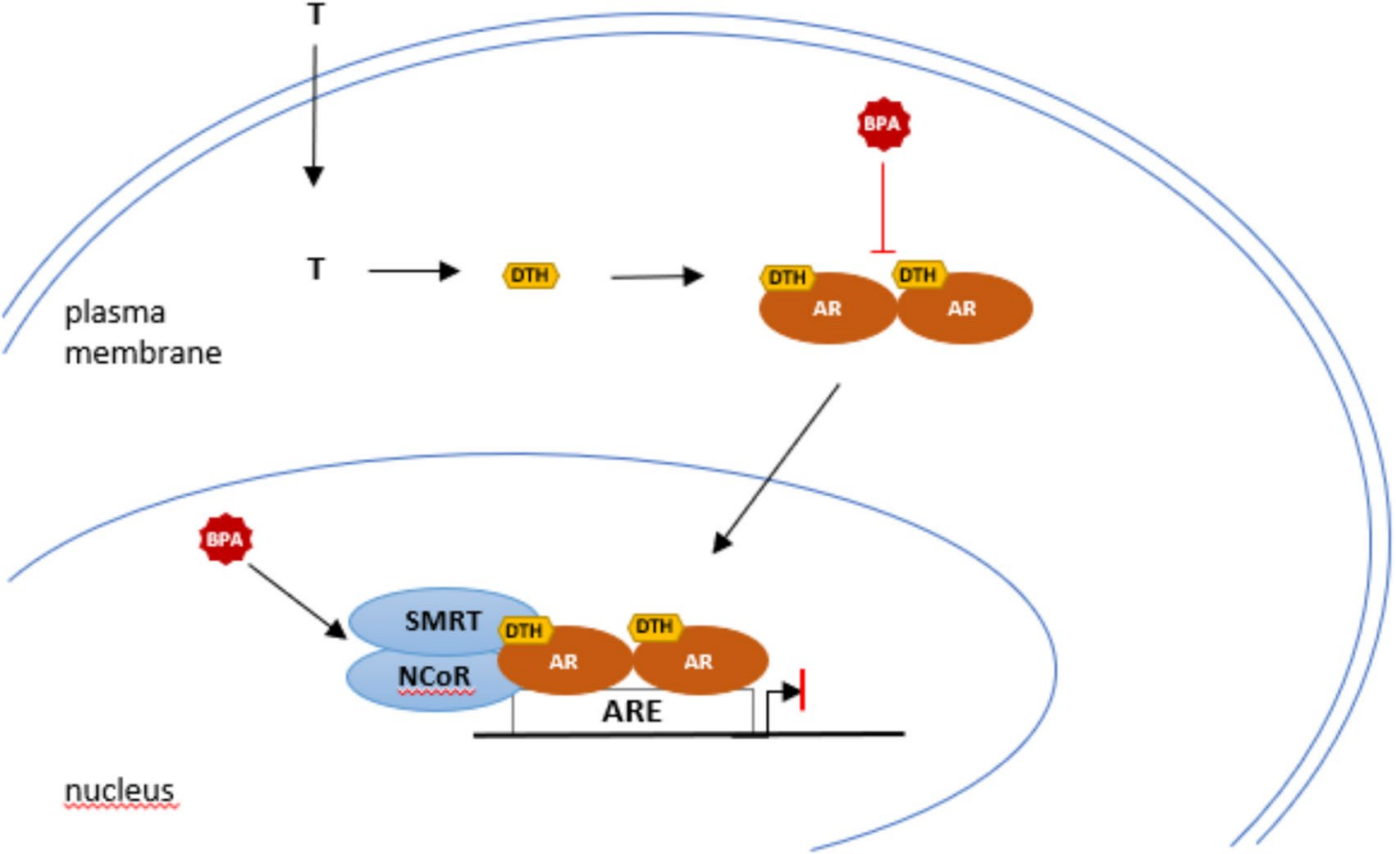
BPA anti-androgenic mechanisms. BPA functions as an AR antagonist by exhibiting inhibitory effects on AR-N/C interaction and dimerization of AR and by enhancing interactions with the corepressors AR-SMRT and AR-NCoR. *T* testosterone; *AR* androgen receptor; *BPA* bisphenol A; *DHT* dihydrotestosterone; *ARE* androgen response element; *N/C* amino- and carboxyl-terminal regions; *SMRT* silencing mediator of retinoic acid thyroid hormone receptor; *NCoR* nuclear receptor co-repressor

Additional anti-androgenic mechanism has been proposed by which BPA antagonizes AR signaling (Fig. 5) (Wang et al. 2017). In addition to the processes related to the interaction between androgen receptors and nuclear receptors, the involvement of co-regulators in the antiandrogenic effects of BPA may also be important. The assembly of the functional AR transcriptional complex requires the interaction between the AR and its co-regulators (Heemers and Tindall 2007). Thus, the transcriptional activities of the AR are mediated by co-regulators such as co-activators and co-repressors. It is assumed that the co-activators bind to the AR and enhance the activation of the receptor. Steroid receptor coactivator-1 (SRC-1) was the first coactivator to be identified within the steroid receptor superfamily (Oñate et al. 1995). However, corepressors also play a role in the repressive function of the AR (Mottis et al. 2013). Two very well-studied transcriptional corepressors are the Silencing Mediator of Retinoic Acid and Thyroid Hormone Receptor (SMRT) and the Nuclear Receptor Co-Repressor (NCoR). It was found that the SMRT suppresses the transactivation of the AR (Liao et al. 2003). BPA acts as an AR antagonist by inhibiting contact between the AR and its co-activator proteins, while promoting interactions between the AR and its co-repressor proteins SMRT and NCoR at environmentally relevant concentrations (40 µM) (Wang et al. 2017).

In contrast to previously described interactions of BPA with nuclear receptors, BPA and analogs do not activate the AR by binding to the binding site, but act antagonistically by binding to the N/R terminus of the AR and interacting with the co-repressors NCoR and SMRT. For a better understanding of the mechanism, it is therefore crucial to investigate the way in which BPA and its analogs interact with the above-mentioned co-repressors.

### Influence of BPA and its analogs on thyroid activity

Thyroid hormones (THs) play a crucial role in the differentiation, growth, metabolism and physiologic function of virtually all tissues (Yen 2001). Due to its structural similarity to TH, BPA also has the potential to bind to the thyroid receptor (TR). BPA has been shown to be a competitive inhibitor of TR and to disrupt TR-mediated gene transcription in vitro and in vivo (Moriyama et al. 2002; Terrien et al. 2011). However, in vitro studies show that BPA binds to both TR-α and TR-β with relatively low affinity. The suppressive effect of BPA on TR transcription is probably not caused by competition with TH for TR binding. Furthermore, it was shown that the suppression of TR transcription is not due to dissociation of the coactivator SRC-1 from TR either in vivo or in vitro. The TR transcription suppression of low concentration BPA was due to the recruitment of corepressors NCoR or SMRT to TR-β1 (Sheng et al. 2012), the proposed mechanisms by which BPA also antagonizes AR signaling (Wang et al. 2017).

**Fig. 6:**
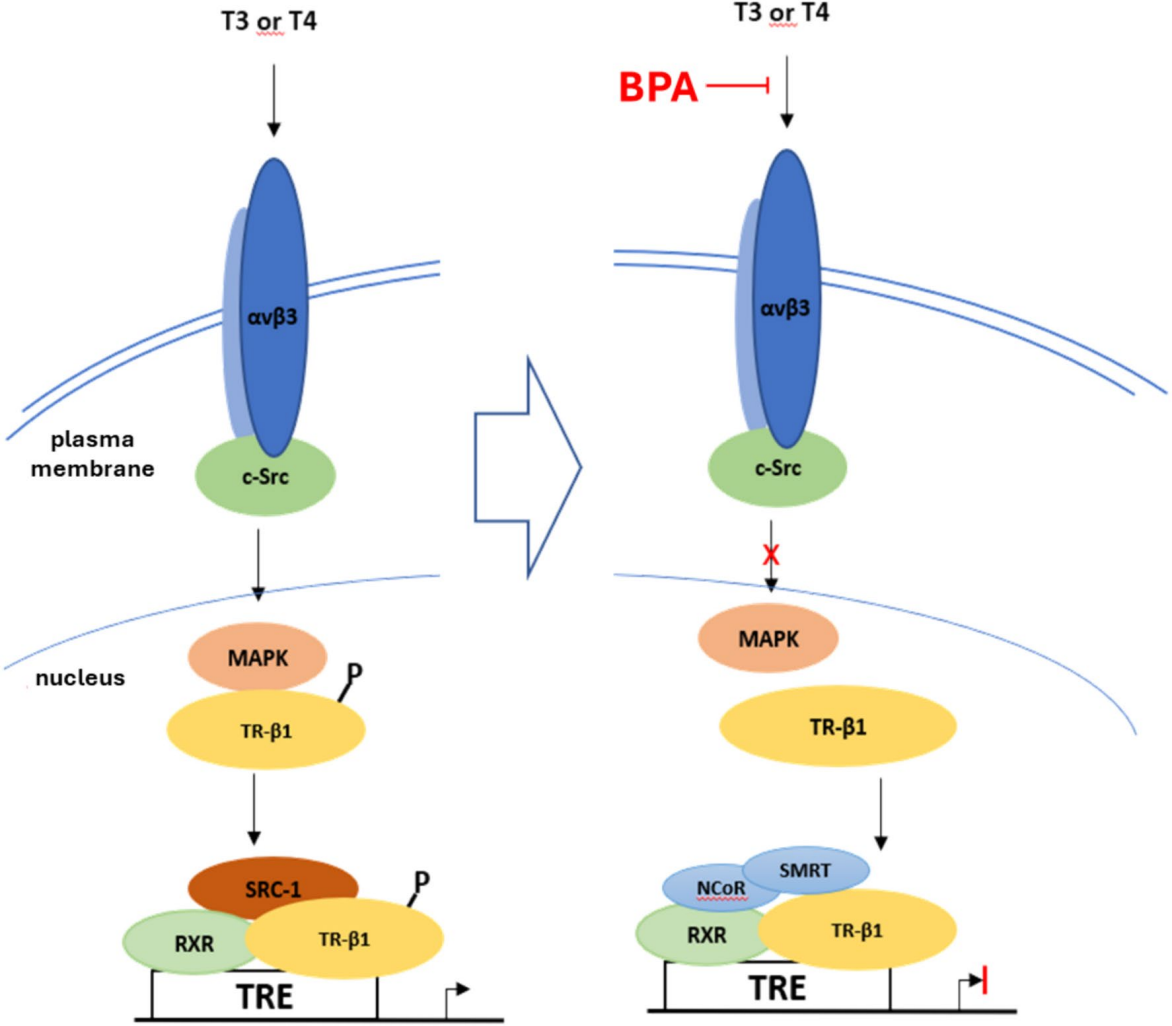
A proposed mode by which low concentrations of BPA suppress the TR transcription (adapted from Sheng et al. 2012). BPA suppressed TR-mediated transcription through recruiting the NCoR or SMRT to TR, which was accomplished by disrupting the TH-regulated integrin αvβ3/c-Src/MAPK/TR-β1. *SRC-1* steroid receptor coactivator-1; *SMRT* silencing mediator of retinoic acid thyroid hormone receptor; *RXR* retinoid X receptor; *NCoR* nuclear receptor co-repressor; *MAPK* mitogen-activated protein kinase; *αvβ3* integrin αvβ3, *C-Src* proto-oncogene tyrosine-protein kinase Src; *TRE* thyroid response element

The classical molecular mechanism of TH action involves uptake of TH by target cells, access of T3 to the cell nucleus and complex formation of the hormone with the nuclear TR, which sheds corepressors after binding of T3 and recruits co-activators to the thyroid response element (TRE) and subsequent hormone-responsive gene transcription, i.e., a genomic pathway (Yen et al. 2006). However, effects of TH have also been described in a variety of cells that do not primarily involve the nuclear TR (Davis et al. 2008) and are thus ‘non-genomic'. It is known that the non-genomic effects of TR depend, at least in part, on the integrin αvβ3 and the cellular signal transduction systems it regulates (Bergh et al. 2005; Davis et al. 2008). T3 and T4 are agonists of integrin αvβ3 (Bergh et al. 2005) and cause activation of mitogen-activated protein kinase (MAPK) by the proto-oncogenic tyrosine-protein kinase Src (c-Src). The activated MAPK can then translocate into the cell nucleus and phosphorylate the nuclear TR-β-DBD (Ser-142). This leads to an altered transcriptional activity of the receptor through the release of the co-repressor proteins NCoR and/or SMRT and the recruitment of the co-activators SCR-1 (Davis et al. 2008). On the other hand, BPA induces integrin αvβ3/c-Src/MAPK/TR-β1 pathways involved in the recruitment of NCoR or SMRT to TR-β1, resulting in the TR transcription suppression. Therefore, it is likely that inactivation of TR-β1 by BPA through a non-genomic pathway significantly suppressed TR transcription, in contrast to the effects induced by acting as an antagonist (Fig. 6) (Sheng et al. 2012).

In addition, Hu et al. have shown that BPA analogs interfere with the pathway of TH synthesis, leading to thyroid dysfunction in mice. It was observed that BPA analogs (BPA, BPS, TBBPA and TBBPS) downregulated the expression of thyrotropin receptor (TSHR), sodium iodide symporter (NIS), thyroglobulin (TG) and thyroperoxidase (TPO) in vivo at doses of 0.02 mg/kg body weight/day and 20 mg/kg body weight/day respectively (Hu et al 2023). The thyrotropin (TSH)/TSHR signaling pathway has been shown to regulate thyroid follicular architecture genes and TH expression (Postiglione et al. 2002). In the hypothalamic-pituitary-thyroid axis, thyrotropin-releasing hormone (TRH) is secreted by the hypothalamus. When the pituitary gland is stimulated by TRH, it releases TSH, which binds to TSHR in the follicular epithelial cells of the thyroid gland and thus regulates the growth and differentiation of these cells (Dumont et al. 1992; Latif et al. 2009). Within the thyroid follicular cells, iodide is actively transported by the sodium iodide symporter (NIS) and catalyzed by thyroperoxidase (TPO) (Carvalho and Dupuy 2017). Thyroglobulin (TG), the precursor of thyroid hormones (THs), serves as a protein substrate for iodination (Citterio et al. 2019; Riesco-Eizaguirre and Santisteban 2006). Consequently, iodination of TG initiates the biosynthesis of THs (Citterio et al. 2019; Coscia et al. 2020). Previous studies in rats reported a decrease in gene expression of TSHR, TPO and NIS after BPA treatment (Mohammed et al. 2020). The inhibitory effect of BPA analogs on NIS, TPO and TG could potentially lead to insufficient iodide supply and hinder TH synthesis. It is noteworthy that TBBPA and TBBPS showed a stronger inhibitory effect on the expression of TSHR, TG and TPO compared to BPA and BPS, suggesting a possible inhibition of TH synthesis. In summary, these results suggest that BPA and its analogs can also induce thyroid dysfunction by altering the protein expression of TSHR, NIS, TPO and TG (Hu et al. 2023).

Based on this information, we can conclude that BPA and its analogs downregulate AR and TR through the involvement of co-repressors (NCoR and/or SMRT). While the suppression of TR by BPA via integrin αvβ3 has been shown (Sheng et al. 2012), this relationship has not been demonstrated for AR. However, it has been suggested that integrin αvβ3 enhances AR transactivation via stimulating JNK1 after ligand binding, which in turn controls AR nuclear traffic (Lu et al. 2016). Therefore, we can assume that AR is similarly downregulated by BPA downregulation of integrin αvβ3.

### Influence of BPA and its analogs on glucocorticoid activity

**Fig. 7:**
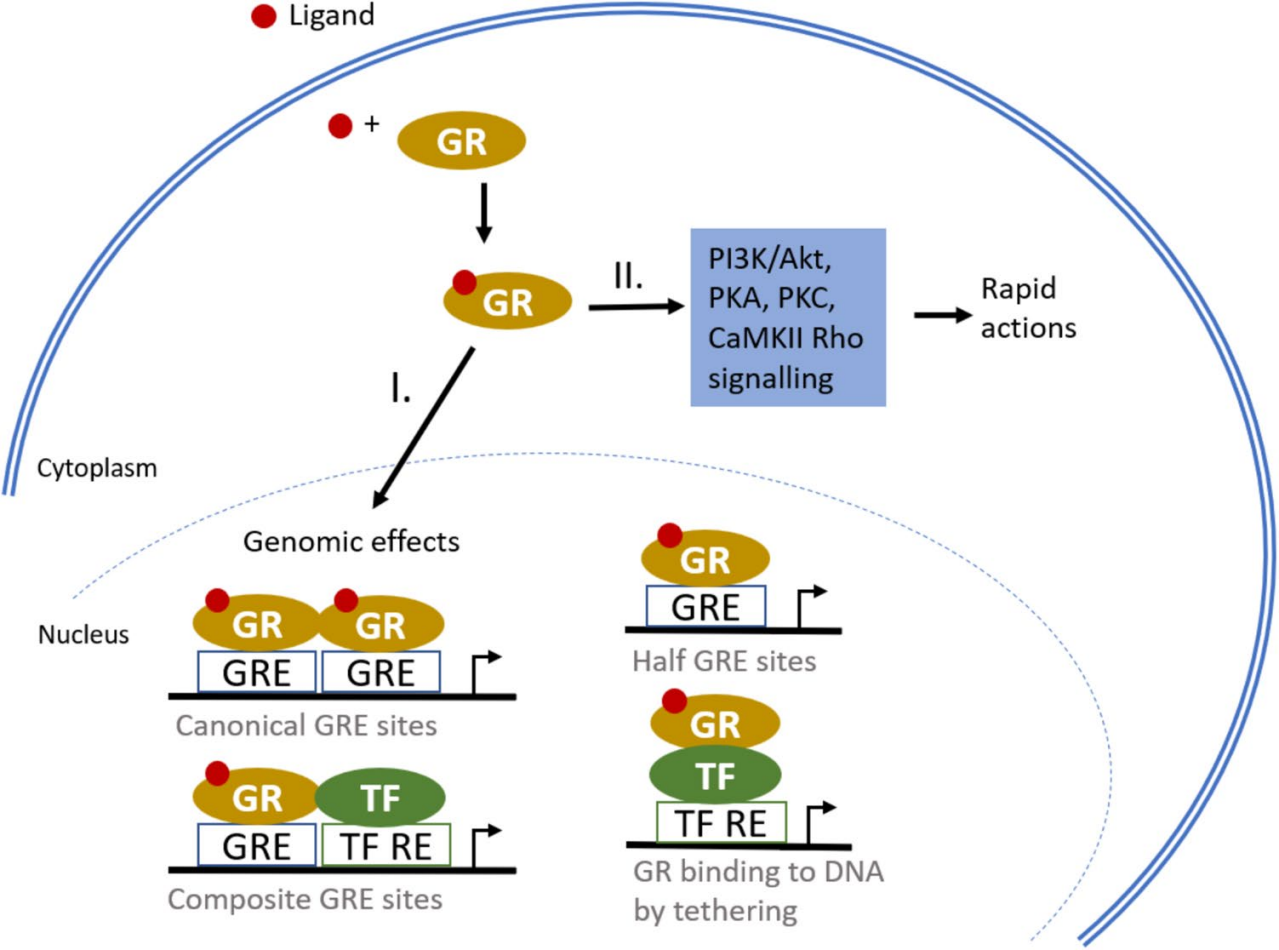
A proposed mode by which low concentrations of BPA suppress the TR transcription. I. The direct genomic signaling pathway is the conventional route of GR-mediated signaling. It involves the direct binding of GR to GRE sequences, either alone or in conjunction with TF. II. The non-genomic signaling pathway is initiated by the binding of a ligand to GR located in the plasma membrane, which leads to the activation of several protein kinase cascades. *GR* glucocorticoid receptor; *TF* transcription factor; *RE* response element; *GRE* glucocorticoid response element; *PI3K* phosphoinositide 3-kinase; *Akt* protein kinase B; *PKA* protein kinase A; *PKC* protein kinase C; *CaMKII* calcium/calmodulin-dependent protein kinase II

The glucocorticoid receptor (GR) is found in almost all types of human cells. GR has many isoforms that function as ligand-dependent TFs via both genomic and non-genomic mechanisms. Binding of GR to the glucocorticoid response element (GRE) can occur either directly or indirectly via other TFs (so-called tethering), and GR action is highly dependent on the GRE type (Scheschowitsch et al. 2017). The primary genomic process involves the activation of the GR by a ligand and the subsequent binding of the GR homodimer to the GRE. GR can also interact with a second transcription factor that binds either on composite biding sites or by tethering. Tethering refers to the indirect binding of GR to DNA, whereby GR binds to TFs that are already bound to DNA. These interactions result in modulation of gene transcription, which is either increased or decreased depending on the specific sequence of the GRE and the promoter region (Newton 2000). In addition, GR is also involved in fast non-genomic signaling pathways, such as PI3K/Akt, PKA, PKC, calcium/calmodulin-dependent protein kinase II (CaMKII)/Rho, which are activated at the cell surface by membrane-bound or cytoplasmic GR (Fig. 7) (Butz and Patócs 2022).

In silico studies have shown that BPA binds to the GR with interaction modes and binding energy comparable to those of traditional GR agonists such as dexamethasone and cortisol, suggesting an agonistic role of BPA at the GR (Prasanth et al. 2010). This prediction is supported by a significant increase in GR-mediated luciferase expression in 3T3-L1 preadipocytes treated with BPA (1 μM) (Sargis et al. 2010). However, in a human breast cancer model MDA-kb2, in which the AR antagonist flutamide was present, BPA did not increase luciferase activity, whereas its analog BPF did. Since both AR and GR are expressed in MDA-kb2 cells and therefore chemicals that bind to either receptor induce luciferase activity, the presence of an AR antagonist is critical to assess only the GR-agonistic activity of the chemicals. Conversely, BPZ and BHEPS showed antiglucocorticoid activity. Although BHEPS exhibited weak activity, BPZ inhibited hydrocortisone-induced luciferase in a concentration-dependent manner with an IC50 of 22 μM (Kolšek et al. 2015). Similarly, Kojima et al. observed no agonistic activity for BPA or eight of its analogs. Six compounds, including BPA, acted as GR antagonists in the order BPAF, BPP > BPAP > BPB, BPZ, BPA. The RIC20 values (20% relative inhibitory concentration) for BPAF and BPP, the most potent GR antagonists among these compounds, were 1.6 µM and 1.7 µM, respectively, which is about three times more potent than BPA. Comparing the RIC50 values (50% relative inhibitory concentration), it was estimated that the GR antagonistic activities of BPAF and BPP are about 30-fold lower than that of RU-486, a known GR antagonist (Kojima et al. 2019).

In addition, adolescent rats exposed to low doses (40 mg/kg body weight per day) of BPA during the perinatal period show changes in their basal and stress-induced function of the hippocampus-hypothalamus–pituitary–adrenal (HHPA) axis in a sexually dimorphic manner. In female rats, there is an increase in basal corticosterone secretion, a less efficient late stress response, and a decrease in hypothalamic GR, accompanied by anxiety as a behavioral trait. In contrast, male rats show a better stress response compared to control animals (Panagiotidou et al. 2014; Poimenova et al. 2010). Children exposed to higher levels of BPA pre- and postnatally (highest quartile compared to the lowest three quartiles) also show depression and anxiety-like behaviors with gender-specific expression patterns (Braun et al. 2011; Harley et al. 2013).

Although the molecular mechanisms underlying these observations in humans are not yet fully understood, studies in adolescent male rat offspring of females exposed daily to 40 μg BPA/kg body weight during pregnancy and lactation have revealed epigenetic modifications. These modifications involve one of the GR co-regulators, FK506-binding protein 5 (FKBP5), a product of the *fkbp5* gene (Kitraki et al. 2015). FKBP5 inhibits the glucocorticoid binding affinity and nuclear translocation of GR in mammalian cells (Wochnik et al. 2005). In cells, a negative feedback loop functions between the GR and its co-regulator FKBP5: the GR activates the transcription of fkbp5, and the translated FKBP5 deactivates the GR by trapping it in the cytoplasm. This process increases resistance to further activation by glucocorticoids (Denny et al. 2000; Scammell et al. 2001). In addition, chronic exposure to glucocorticoids leads to persistent altered FKBP5 expression in the hippocampus and hypothalamus of mice due to changes in DNA methylation of the gene (Lee et al. 2010; Yang et al. 2012). In the study with juvenile rats, BPA exposure led to an increase in DNA methylation of *fkbp5* in the hippocampus and a decrease in FKBP5, a process in which GR is involved. However, the effect of BPA on FKBP5 was abolished upon knock-down of ERβ, suggesting a role for this receptor in mediating the effect of BPA on FKBP5 (Kitraki et al. 2015). These findings not only suggest a potential role for FKBP5 in stress- and mood-related pathologies involving GR, but also link endocrine-disrupting chemicals such as BPA (and its analogs) to glucocorticoid insensitivity and mood disorders (Gulliver 2017).

### Endocrine disruption of BPA and its analogs mediated through peroxisome proliferator-activated receptor

Peroxisome proliferator-activated receptors (PPARs) are ligand-activated TFs that belong to the nuclear hormone receptor superfamily and consist of three subtypes: PPARα, PPARγ and PPARβ/δ. Activation of PPAR-α lowers triglyceride levels and contributes to the regulation of energy balance. Activation of PPAR-γ promotes insulin sensitization and improves glucose metabolism, while activation of PPAR-β/δ improves fatty acid metabolism. Overall, the PPAR family of nuclear receptors is of central importance for the regulation of energy balance and metabolic functions (Tyagi et al. 2011). The interactions between BPA analogs and PPARγ are the best studied. It has been shown that BPS and BPA can activate PPARγ and promote binding to the PPARγ response element (PPRE). However, BPS, but not BPA, was able to competitively inhibit PPARγ activated by the known ligand rosiglitazone (ROSI), suggesting that BPS interacts differently with PPARγ than BPA (Ahmed and Atlas 2016). Similarly, several studies have shown that the agonistic activity of the halogenated analogs of BPA TBBPA and TCBPA toward PPARγ was higher than BPA (Fang et al. 2015; Riu et al. 2011).

The binding of BPA and its halogenated derivatives (halogenated BPAs) to the mouse PPARα ligand binding domain (mPPARα-LBD) was investigated by Zhag et al. BPA and all eleven halogenated BPAs (BPC, BPAF, TBBPA, TCBPA, 1,1,1-trichloro-2,2-bis(4-hydroxyphenyl)ethane (HPTE), 3-monobromobisphenol A (monoBBPA), 3,3′-dibromobisphenol A (diBBPA), 3,3′,5-tribromobisphenol A (triBBPA), 3-monochlorobisphenol A (monoCBPA), 3,3′-dichlorobisphenol A (diCBPA) and 3,3′,5-trichlorobisphenol A (triCBPA)) showed dose-dependent binding to PPARα, resulting in activation of the receptor, which in turn would have a negative effect on physiologic processes. TBBPA, with the maximum number of Br-substituents on the phenol rings, showed the strongest binding to PPARα. However, with the maximum number of F substituents on the bridging alkyl group, BPAF showed the weakest receptor binding (Zhang et al. 2018).

The investigation of the effects of seven BPA analogs (BPA, BPS, BPAF, BPF, BPB, TBBAP, TCBPA) on the PPARβ/δ receptor pathway revealed a similar outcome. The majority of BPA analogs exhibited a dose-dependent increase in luciferase transcriptional activity in the gene- reporting assay, indicating their potential agonistic effect on the PPARβ/δ signaling pathway, with the exception of BPS, which exhibited no PPARβ/δ agonistic activity. While TBBPA and TCBPA showed almost the same agonistic activity as BPA, BPAF, BPF and BPB showed stronger agonistic activity than BPA. Nevertheless, the above findings contradict the results of the binding test. This inconsistency suggests that the agonistic effect of a bisphenol analog against PPARβ/δ may depend on both its binding configuration and affinity (Li et al. 2021). In addition, cellular uptake of BPA analogs may influence their agonistic effect on PPARβ/δ. This may explain the lack of agonistic effect of BPS on PPARβ/δ, as hydrophobicity (LogKow), a key factor for cellular uptake, is lowest for this compound (Li et al. 2020).

Based on these data, a thorough investigation of the relationship between the toxicity of BPA analogs and cellular uptake would be beneficial. Since BPA and its analogs may have different mechanisms of action, testing for endocrine-disrupting properties is time-consuming. Therefore, a simple screening method, such as measuring hydrophobicity, would be useful for rapid screening of new BPA analogs.

### Modulation of pregnane X receptor with BPA and its analogs

The pregnane X receptor (PXR) is a regulator of the expression of genes involved in all phases of drug metabolism and excretion. It induces the expression of CYP450 genes, genes encoding UDP-glucoronosyltransferases and glutathione S-transferases, as well as drug efflux pumps such as multidrug resistance 1 and multidrug resistance protein 2 (Orans et al. 2005). On the other hand, PXR has evolved several structural features that allow it to function as a broad chemical “sensor”. The unique composition of the ligand pocket not only allows PXR to bind a variety of chemicals but also allows a single ligand to dock in multiple orientations. This binding mode stands in sharp contrast to other nuclear receptors, which are highly selective for their cognate hormones (Watkins et al. 2001).

When different BPA analogs were tested for their ability to activate the human PXR (hPXR) using HeLa reporter cell lines, only BPA and its halogenated derivatives, TCBPA and TBBPA, were found to be weak to moderate hPXR activators. TCBPA was the most potent of these compounds and activated hPXR with an EC50 of 8.49 μM. In contrast, BPS and BPF were unable to activate hPXR at concentrations up to 10 μM (Molina-Molina et al. 2013).

In contrast, Muse PXR or rat PXR were not activated by BPA in transfection assays (Sui et al. 2012), suggesting a species-specific response. This was further demonstrated by Siu et al. who created a PXR-humanized ApoE-deficient (huPXR-ApoE−/−) mouse line that responds to human PXR ligands and performed feeding experiments to determine the effects of BPA exposure on the development of atherosclerosis. The area of atherosclerotic lesions of huPXR-ApoE−/− mice was significantly increased by 104% (*P* < 0.001) and 120% (*P* < 0.05) after BPA exposure. In contrast, BPA had no effect on the development of atherosclerosis in littermates without human PXR (Sui et al. 2014). This raises an important question, as the choice of an appropriate animal model is crucial for predicting the risk assessment of BPA in humans.

## Conclusion

The phasing out of bisphenol A (BPA) in industry has led to the widespread use of structurally similar BPA analogs. New evidence suggests that many of these compounds have comparable or even stronger endocrine-disrupting effects. Several different BPA analogs have been shown to interact with multiple nuclear and membrane receptors. These interactions lead to changes in downstream signaling pathways such as Ca2 + signaling, the PI3K/Akt and ERK/MAPK cascades, as well as epigenetic modifications that may enhance their toxic effects.

In particular, BPAF and BPB show stronger ERα- and ERβ-agonistic activity than BPA, while BPS, BPAF and TCBPA exhibit significant activation of GPER, which promotes cancer cell proliferation. BPA analogs such as BPB and TBBPA also show higher affinity for PPARs, which are important regulators of lipid metabolism and glucose homeostasis. In addition, certain analogs such as chlorinated BPC show a pronounced anti-androgenic effect, while BPAF and BPB act as potent GR antagonists.

Despite these findings, there remain significant gaps in our understanding of how BPA analogs disrupt endocrine function at environmentally relevant concentrations. Further research is needed to assess their potential synergistic and additive effects that could exacerbate endocrine disruption. In addition, studies should prioritize the mechanisms of cellular uptake, as hydrophobicity and membrane permeability can significantly influence toxicokinetics. Furthermore, species-specific differences in toxicological responses emphasize the need for models relevant to humans to improve the accuracy of risk assessment.

Considering that BPA analogs interfere with multiple signaling pathways, regulatory assessments should go beyond the binding affinity of nuclear receptors as the main criterion for safety assessment. Instead, comprehensive toxicity profiling should incorporate alternative mechanisms of endocrine disruption, including epigenetic changes, interactions with membrane receptors, and overlap with other endocrine systems. Without such precautions, replacing BPA with structurally similar compounds could exacerbate rather than mitigate risks to public health and the environment.

Future studies should focus on long-term epidemiologic data, mechanistic insights into the effects of low doses, and the identification of novel, safer alternatives to bisphenols that minimize endocrine-disrupting potential.

## Data Availability

All data produced or analyzed during this study are enclosed in this article.
